# Atomic Force Microscopy Analysis of the *Acinetobacter baumannii* Bacteriophage AP22 Lytic Cycle

**DOI:** 10.1371/journal.pone.0047348

**Published:** 2012-10-11

**Authors:** Evgeniy V. Dubrovin, Anastasia V. Popova, Sergey V. Kraevskiy, Sergei G. Ignatov, Tatyana E. Ignatyuk, Igor V. Yaminsky, Nikolay V. Volozhantsev

**Affiliations:** 1 M.V. Lomonosov Moscow State University, Moscow, Russian Federation; 2 Advanced Technologies Center, Moscow, Russian Federation; 3 State Research Center for Applied Microbiology and Biotechnology, Obolensk, Russian Federation; 4 Alikhanov Institute for Theoretical and Experimental Physics, Moscow, Russian Federation; Charité-University Medicine Berlin, Germany

## Abstract

**Background:**

*Acinetobacter baumannii* is known for its ability to develop resistance to the major groups of antibiotics, form biofilms, and survive for long periods in hospital environments. The prevalence of infections caused by multidrug-resistant *A. baumannii* is a significant problem for the modern health care system, and application of lytic bacteriophages for controlling this pathogen may become a solution.

**Methodology/Principal Findings:**

In this study, using atomic force microscopy (AFM) and microbiological assessment we have investigated *A. baumannii* bacteriophage AP22, which has been recently described. AFM has revealed the morphology of bacteriophage AP22, adsorbed on the surfaces of mica, graphite and host bacterial cells. Besides, morphological changes of bacteriophage AP22-infected *A. baumannii* cells were characterized at different stages of the lytic cycle, from phage adsorption to the cell lysis. The phage latent period, estimated from AFM was in good agreement with that obtained by microbiological methods (40 min). Bacteriophage AP22, whose head diameter is 62±1 nm and tail length is 88±9 nm, was shown to disperse *A. baumannii* aggregates and adsorb to the bacterial surface right from the first minute of their mutual incubation at 37°C.

**Conclusions/Significance:**

High rate of bacteriophage AP22 specific adsorption and its ability to disperse bacterial aggregates make this phage very promising for biomedical antimicrobial applications. Complementing microbiological results with AFM data, we demonstrate an effective approach, which allows not only comparing independently obtained characteristics of the lytic cycle but also visualizing the infection process.

## Introduction

*Acinetobacter baumannii* is a nosocomial pathogen, which is often associated with hospital-acquired infections such as pneumonia, wound and urinary tract infections, post-surgery complications and bloodstream infections, especially in severely ill and immunocompromised patients of intensive care and burn units [1], [2]. The microorganism is characterized by its widespread resistance to the major groups of antibiotics, as well as to disinfectants and detergents and was shown to spread and survive in health care environments and on medical devices [3]–[5]. This remarkable resistance to bactericidal factors could be associated with the formation of *A. baumannii* biofilm on various biotic and abiotic surfaces. Thus, eradicating *A. baumannii* from hospital settings remains currently a challenging task.

Because of the lack of effective therapeutic agents, novel antimicrobials, which might be active against *A. baumannii,* are urgently needed. The application of lytic bacteriophages is a potential solution to this problem. Recently, several lytic bacteriophages infecting *A. baumannii* clinical strains have been characterized [6]–[9]. However, research focusing on *A. baumannii* infection by specific virulent bacteriophages and investigations of different phases of the phage infection process are still lacking.

Studies of bacteriophages and their infection of host cells benefit from using direct methods of investigation, since they provide important information which complements microbiological data. Atomic force microscopy has already become a widespread tool for studying microbial surfaces (for reviews, see [10]–[11]). Comparably easy sample preparation procedures, ability to operate in ambient or aqueous conditions, possibility to obtain 3D topographical images and evaluate mechanical properties of the sample under investigation make AFM the most efficient tool for studying bacteriophages and infected bacterial cells among the other high resolution types of microscopy, such as electron microscopy (EM) methods. AFM allows investigating specific interaction at the level of a single bacterial cell or even less [12]. AFM was used for the investigation of phages adsorption on the surfaces under different conditions [13]–[16]. For example, it was shown that PEG modification of T4 bacteriophages leads to their aggregation on the mica surface [13] and adsorption of phages to the surface may be affected by pH value [14], underlying pattern [15] or type of environment [16]. The stiffness of bacteriophage capsid as a function of packaged genome length and ionic environment was studied using AFM nanoindentation experiments [17]–[18]. Advantages of AFM over transmission electron microscopy (TEM) for investigation of extruding filamentous phages from *E. coli* cells were reported by M. Ploss and A. Kuhn [19]. Higher number of extruding phages observed by AFM was attributed to the rupture of some of them during sample preparation for TEM. Recently, we have developed investigation protocols for bacteriophage infection of bacterial cells using AFM [20]. This approach allows monitoring different stages of the process of infection, from phages adsorption on the cell surface to the release of new born phages and destruction of the host cell. Different aspects of bacteriophage infection of *E. coli*, *S.* Enteritidis, *B. thuringiensis* strains and some pelagic bacteria were studied using AFM [19]–[22].

In the present study, we have performed the analysis of the *A. baumannii* phage AP22 lytic cycle and AFM visualization of the interaction between the phage and host bacterial cells. AFM was also used to analyze the surface of *A. baumannii* cells and their phages, formation of bacterial aggregates and their dispersal during phage infection.

## Results

### Microbiological Assessment of Phage AP22 Infection

Bacteriophage AP22 efficiently lysing clinically relevant multidrug-resistant *A. baumannii* strains has been recently described. According to TEM, the phage was found to be a representative of the *Myoviridae* family with an icosahedrical head of 64 nm in diameter and a contractile tail of 85–90 nm in length [9].

**Figure 1:**
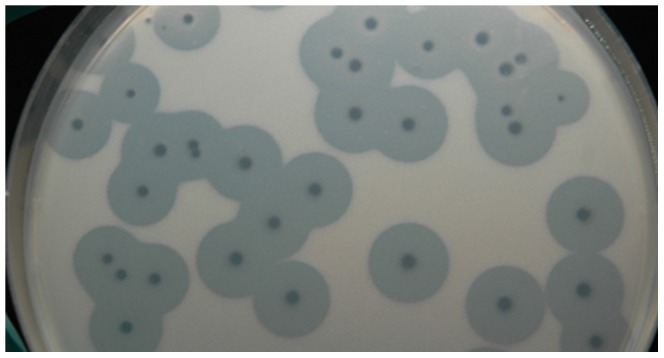
Phage AP22 plaques. Phage AP22 plaques with opaque haloes on the host *A. baumannii* 1053 strain.

*A. baumannii* 1053 strain forming mucoid colonies on the agar plates was used as a host for bacteriophage AP22 propagation. The phage formed clear, round plaques surrounded by expanding with time opaque haloes on bacterial lawn of the strain (figure 1). These halo zones can indicate the presence of phage depolymerase capable of degrading exopolysaccharide or extracellular polymeric substances (EPS) secreted by *A. baumannii* mucoid cells [23], [24].

In our previous work the phage AP22 adsorption assay and one-step growth experiments were performed in a phage-bacteria system [9]. Bacteriophage AP22 was shown to have an initial phase of very rapid adsorption to the host bacterium (more than 99% of the phages adsorbed within 5 min) followed by a period of slower adsorption. One-step growth experiments have demonstrated a 40-min latent period and a burst size of approximately 240 phage particles per one infected bacterial cell [9].

**Figure 2:**
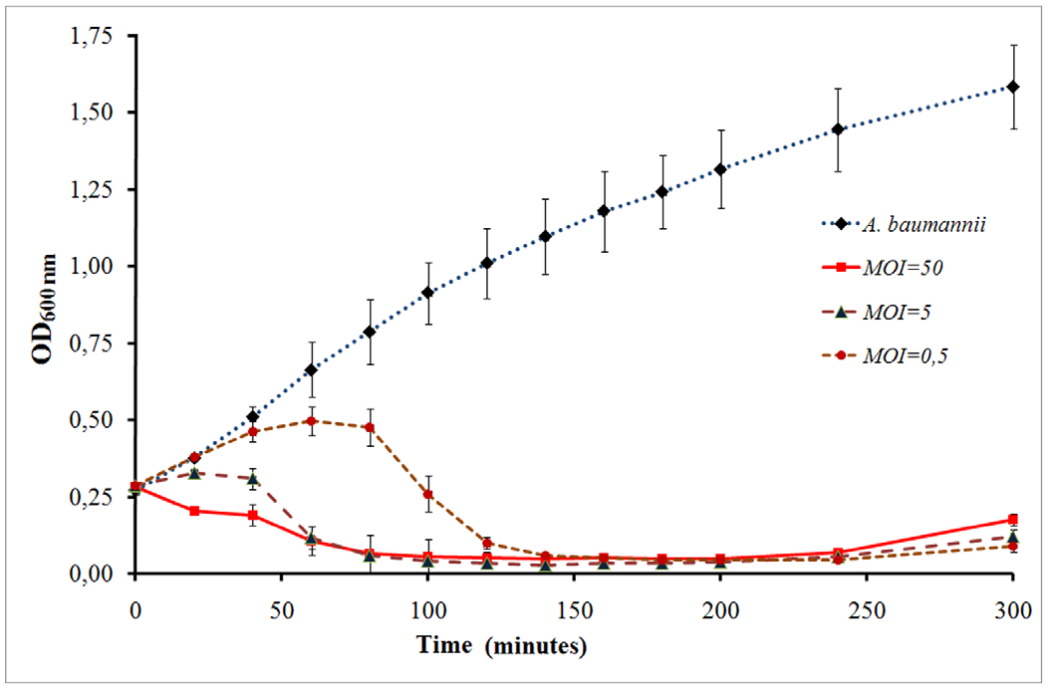
Infection curves of phage AP22. Representative curves are based on results from three independent assays of a non-infected *A. baumannii* culture and cultures infected at MOI of 0.5, 5 and 50. Standard deviations are indicated.

Here, we have performed the experiments, studying the dynamics of phage AP22 infection process by determining the optical density (at 600 nm) of the *A. baumannii* 1053 infected cells at different times. These measurements were carried out at three different multiplicities of infection (MOI, the ratio of phage particles to host cells), including that used in our AFM experiments (MOI of 50). Infection curves pictured in the figure 2 suggest phage AP22 completely lyses the strain in approximately 60 minutes at MOI of 50 and 5 and in 120 minutes at MOI of 0.5. After lysis of *A. baumannii* 1053 liquid culture by the phage the optical density at 600 nm remains stable.

### AFM Analysis of *A. baumannii* Bacteriophage AP22

**Figure 3:**
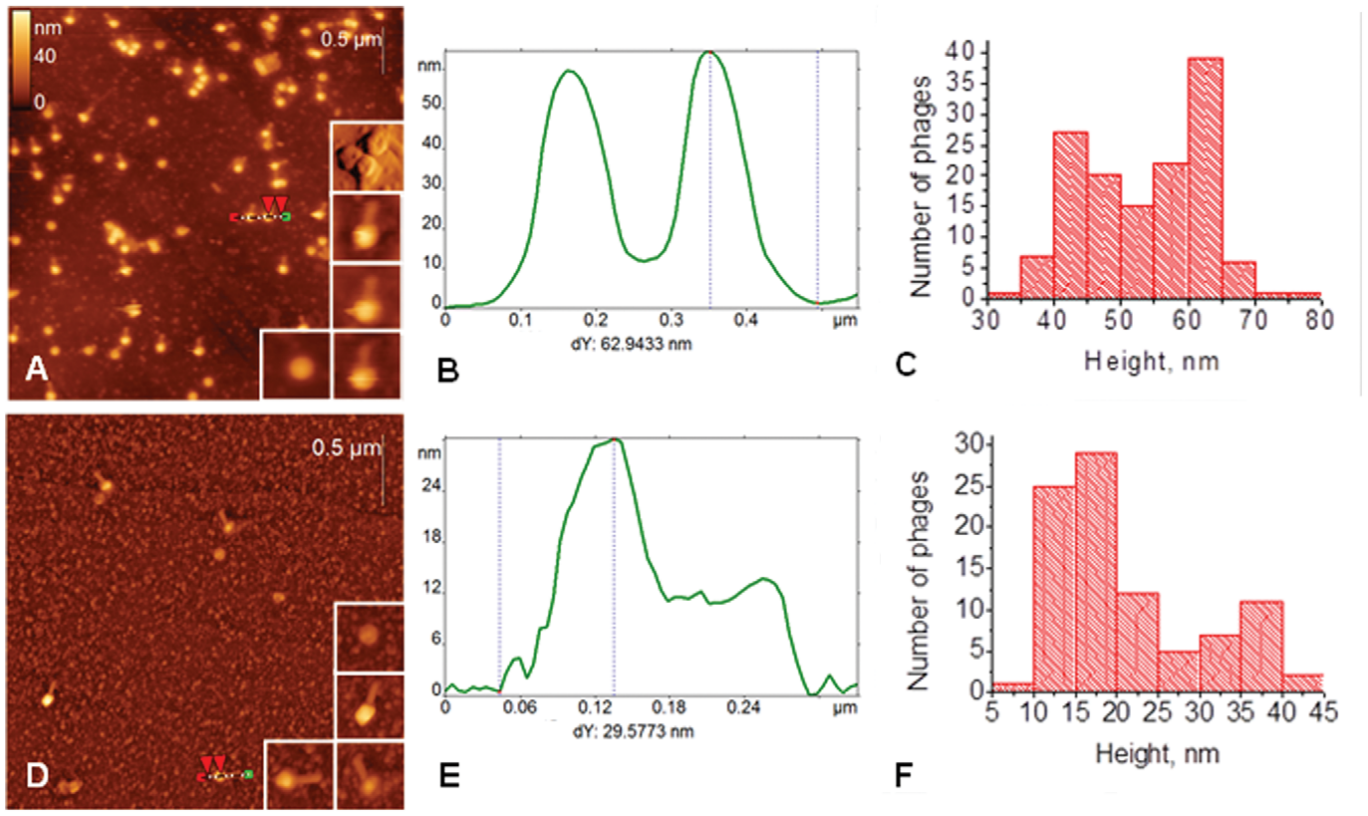
AFM images of bacteriophages AP22. AFM height images (A, D), section analysis (B, E) and height distribution histograms (C, F) of bacteriophages AP22 deposited on the mica (top row) and HOPG (bottom row) surfaces. Sections are built along the dotted lines on the corresponding AFM images. Insets in A and D demonstrate zoomed bacteriophages from the same image. Top inset in A is presented in deflection channel to emphasize holes in the center of the phages.

Phage AP22 deposited on surfaces of mica and highly oriented pyrolytic graphite (HOPG) was examined using AFM, which clearly resolved the head and, in many cases, the tail of this phage (figure 3). AFM images have revealed dramatic difference in the apparent heights of bacteriophages, adsorbed onto these substrates, which is illustrated by the height distribution histograms in figure 3C, F: the heights of bacteriophages heads on HOPG lie within the range of 10–40 nm, whereas this range for mica substrate constitutes 40–70 nm. This phenomenon is probably connected with hydrophobic interaction of the bacteriophage protein head with graphite surface, which leads to the partial destruction of a phage particle. HOPG-induced partial destruction of tobacco mosaic virus was also reported elsewhere [25]–[26]. The shapes of the height histograms presented in figures 3C, F have two maximums, which demonstrate the presence of two states of bacteriophages: with full and (partially) empty heads. Taking into account that phages adsorbed on HOPG may be partly destroyed, we further refer to phage dimensions obtained on mica. Mean height of full bacteriophage heads (second maximum in the figure 3C) constitutes 62±1 nm. This value may be considered as an estimation of the real diameter of the bacteriophage head. The diameter of the phage AP22 head obtained by EM was 63–65 nm [9]. Such good compliance with EM results demonstrates that height measurements are quite accurate in our experiments. Small indentations in the center of empty heads, which correspond to capsids with (partially) released DNA, are often resolvable in AFM images (see upper inset in figure 3A, which was acquired in the deflection channel). The presence of heads with (partially) released DNA after their adsorption on mica was also reported by us for other bacteriophages [20]. Earlier works have reported the decreased rigidity of empty bacteriophages’ capsids [17], [27]. The observed release of DNA from bacteriophage heads may be initiated by osmotic shock during the rinsing stage [27], the drying of the sample [15] or the interaction with the substrate. Since the fraction of empty heads is much higher on HOPG than on mica (figure 3C, F), the latter reason plays bigger role for bacteriophage AP22 adsorption on HOPG. The length of the bacteriophage AP22 tail constitutes 88±9 nm that is in a good agreement with EM data (85–90 nm, [9]).

### AFM Analysis of *A. baumannii* cells and their Aggregates

**Figure 4:**
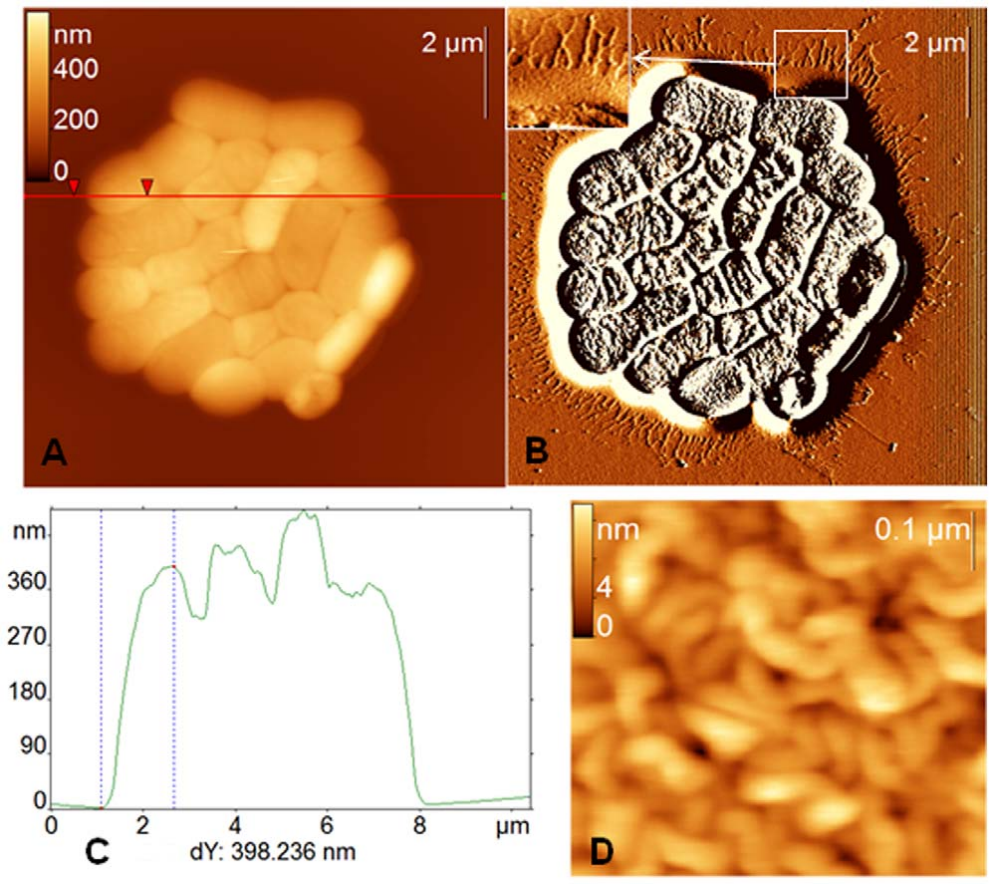
AFM images of *A. baumannii* cells. AFM height (A) and deflection (B) images of *A. baumannii* cells deposited on the mica surface. (C) Section analysis built along the horizontal line in (A). (D) Zoomed-in region of *A. baumannii* surface. The inset in (B) demonstrates zoomed-in region around bacterial aggregate (shown by the white square) with the EPS and pili.

*A. baumannii* cells, grown as described above, adsorb on the mica surface as aggregates comprising several tens of cells or more (typical aggregate is shown in figure 4). The estimation of the volumes of such aggregates from AFM images gives us the values in the range (10–100)×10^−12^µl (for example, the volume of the aggregate presented in the figure 4A is approximately 13×10^−12^µl). These estimations demonstrate that the quantity of such aggregates could be very high even in a 1 µL droplet of a suspension, which we used.

One can see a relatively thin layer about 15 nm in height and 100–200 nm in width together with a very dense network of radial linear structures around *A. baumannii* aggregates (see figure 4B). According to earlier works with similar AFM observations of *A. baumannii* cells we attribute the layer to the protective matrix made of EPS [28] and radial structures to pili [23]. The length of pili measured from the edge of the cells ranges from 0.5 to 1.7 µm and their apparent height on the surface is below 1 nm. The real diameter of pili may be higher due to “height lowering effect” of AFM tip in contact mode.

AFM revealed different sizes (especially the length) of the cells, probably indicating different stages of cell growth. However, their length, width and height may be estimated from AFM images as follows: 1–2 µm (length), 0.75–1 µm (width) and 0.4–0.5 µm (height). The surface of *A. baumannii* cells is zoomed in figure 4D, which demonstrates the typical ripple structure of the bacterial envelope [23]. Its root mean square (RMS) roughness is about 2.5 nm.

### AFM analysis of the *A. baumannii* Bacteriophage AP22 Lytic Cycle

**Figure 5:**
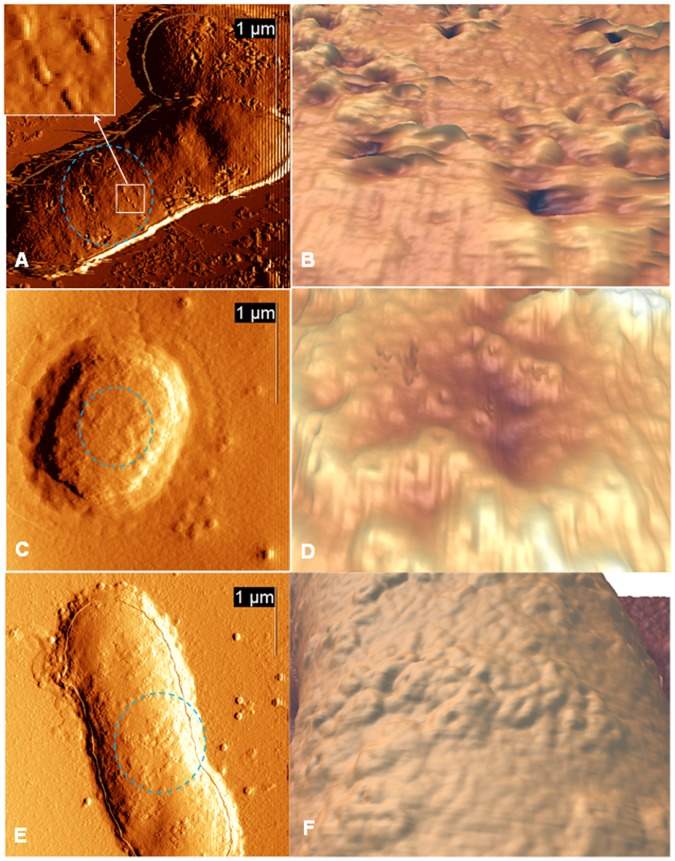
AFM images of *A. baumannii* cells infected for 1–6 minutes. Left row: deflection AFM images of *A. baumannii* cells incubated with bacteriophages AP22 for 1 (A), 3(C), 6 (E) minutes. Right row: zoomed-in three dimensional reconstructions of bacterial surfaces depicted on the left (the zoomed regions are indicated by dotted circles in the height images). The inset in (A) demonstrates zoomed-in region, shown by the white square.

**Figure 6:**
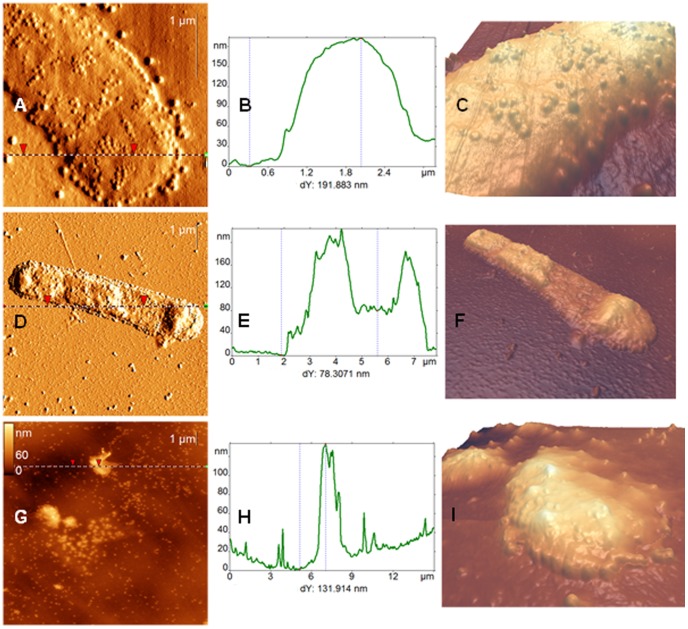
AFM images of *A. baumannii* cells infected for 15–60 minutes. AFM deflection (A, D) and height (G) images of *A. baumannii* cells incubated with bacteriophages AP22 for 15 (A), 30 (D), 60 (G) minutes. (B), (E) and (H) represent the section analysis along the dotted lines on (A), (D) and (G) correspondingly. (C), (F) and (I) – zoomed-in three dimensional reconstructions of bacterial surfaces depicted on the left.

In the next series of experiments we studied the morphology of *A. baumannii* cells, incubated with their phages at 37 °C during different periods of time varying from 1 to 60 minutes. Cells lose their biofilm-like structure after interaction with the phage that can be concluded from the obtained AFM images. In most cases such cells adsorbed onto mica individually and sometimes in pairs (only for one-minute period of incubation) (figures 5–6). Bacteriophage adsorption onto the surface of *A. baumannii* cells was already visible in AFM images after 1 minute of their mutual incubation (figures 5A, B). Obviously, these phages serve as an excellent disaggregation factor (additional AFM image, demonstrating separation of the aggregates at a lower scale is included in figure S1).

The number of adsorbed phages significantly increased during the following 10 minutes of their incubation with the cells (figures 5C–F). In contrast to what had been observed in the experiments on phages adsorption onto mica and graphite surfaces, almost all of adsorbed on the host cells bacteriophages had empty heads. This indicates that DNA release takes place quite fast or probably immediately upon phages adsorption on the host cell. RMS roughness of the cell surface has increased to 3–4 nm for the samples deposited after six minutes of incubation. The height of the cells has not changed significantly within the first minutes of phage-cells incubation.

The results of bacteriophages AP22 incubation with *A. baumannii* cells during 15, 30 and 60 minutes are presented in the AFM images in figure 6. We can see very dense phage adsorption on the cell and, beginning from 30 minutes of incubation, significant changes in the shape of bacterial cells. The apparent height of the cell became inhomogeneous along the cell, varying from 70 to 200 nm (figure 6D–I) that is 2–5 times lower than the cells height measured on the reference AFM images (see figure 4). Variations of the morphology of *A. baumannii* cells infected for 30 minutes are presented in figure S2. 60-minutes incubation leads to the destruction of the cells (that can be concluded from the complete loss of their normal shape and height) and the release of newborn phages from the cell (figures 6G–I, variations are presented in figures S3 and S4). Changes of the shape of infected *A. baumannii* cells are accompanied by the significant increase of RMS roughness of the cell wall surface up to 10 nm and more.

## Discussion

Recently, several other lytic *A. baumannii* phages have been described [6]–[8]. They demonstrated high efficacy of adsorption, short latent periods (not exceeding 20 minutes) and large burst sizes (more than 100 PFU/infected cell). Here, using AFM in complement to microbiological assays, we have investigated *A. baumannii* phage AP22 and its lytic cycle. Phage AP22 exhibited very fast adsorption and ability to completely lyse a liquid culture of the host strain in different time depending on MOI.

Since AFM was utilized for the first time to study specific interaction of bacteriophage with *A. baumannii*, we have used scanning in air as in the methodologically simplest conditions of operation, especially for high resolution imaging. Despite possible artifacts due to drying, information regarding the morphology of biological objects (in our case bacteriophages and infected cells) remains valuable that was demonstrated many times in previous works (e.g., [23], [29]–[32]). Also it should be noted that *A. baumannii* can survive for long periods on dry surfaces [4], [33].

Stagnantly incubated *A. baumannii* cells are able to form biofilms at the air-liquid interface (so called pellicles) [23], [34]–[36]. AFM images of *A. baumannii* pellicles incubated for 24 hours demonstrated big colony islands more than 100 µm in diameter, accompanied by microcolonies in their vicinity [23]. Remarkably, both AFM images of *A. baumannii* big pellicles [23] and small aggregates (figures 4A, B) reveal the presence of the EPS matrix and pili, which were considered to be the attributes of *A. baumannii* biofilm formation [37]. The presence of these attributes together with the use of short incubation time (90 minutes) of *A. baumannii* cells before their deposition onto mica allows suggesting that the observed cells aggregates (figure 4A) are early stage pellicles, or “proto-pellicles”. Formation (or inner reorganization) of these early stage pellicles on the mica surface is unlikely due to a very short time of their adsorption (only 1 minute) and the absence of nutrients.

Biofilm formation is an adaptation mechanism of the cells, which helps them to survive at harsh conditions [37]–[38] and, in particular, resist to antibiotic treatment [39]. Application of lytic phages is one of the most promising approaches for struggling against bacterial pathogens [40]. The lytic cycle of the novel bacteriophage AP22 was characterized using microbiological assessment and visualized by AFM. One-minute bacteriophage treatment of *A. baumannii* aggregates at 37°C has led to an almost complete dispersal of “proto-pellicles”, which was accompanied by phage adsorption on the cell surface (figures 5A, B). Significant surface changes were observed after 30 minutes of mutual incubation of *A. baumannii* cells with the phage. AFM results are in good compliance with the results of the microbiological assessment of infection process, which has also demonstrated the phage AP22 adsorption from the first minute of incubation and a latent period of 40 minutes [9].

Though the bacteriophage induced biofilm destruction was reported quite a lot of times (for review see Ian W. Sutherland et al. [24]), all its mechanisms are not well-known to date. In many cases biofilm dispersal is facilitated by polysaccharide-degrading enzymes which some bacteriophages possess [24]. The very fast “proto-pellicles” dispersal observed in our experiments after adding bacteriophages supposes that phage AP22 possibly possesses polysaccharide depolymerase, which is confirmed by the presence of haloes surrounding the phage plaques on the plates (figure 1).

**Figure 7:**
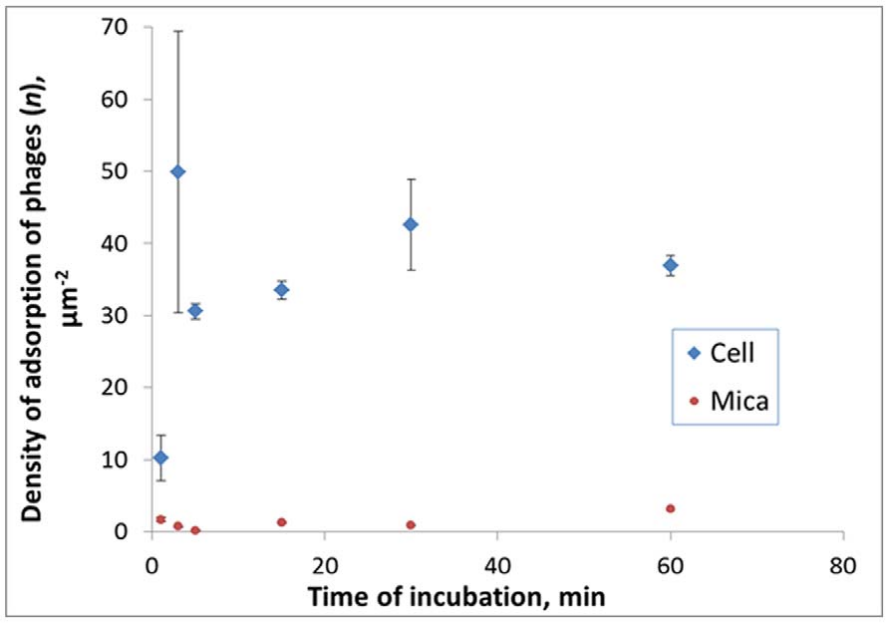
The density of adsorption of bacteriophages AP22. The density of adsorption of bacteriophages AP22 on the surfaces of mica and bacteria for the samples incubated during different time periods.

Since the multiplicity of infection was very high in AFM experiments (MOI of 50), not all phages adsorbed on the surface of the cell, and part of them remained in the solution. That is why we have observed the adsorption of phages both on bacteria and mica. In order to compare the bacteriophage adsorption on these two surfaces we have evaluated the number of phages per square unit (*n*) adsorbed on mica and on bacteria after different times of bacteria incubation with phages (figure 7). The density of phages adsorbed on the host cells was much higher in all cases (for 1, 3, 5, 15, 30 and 60 minutes of incubation). We observe a significant increase of the number of phages adsorbed on the host cell in the first 3 minutes of infection, when the density of adsorption reaches 50±20 particles/µm^2^. This number remained on the similar level (within the errors) at all later stages of the phage infection of bacteria. The level of the number of phages adsorbed on the mica surface was below 2 particles/µm^2^ at all times of incubation except for 60 min period, when the density increased to 3.2 particles/µm^2^. This should be connected with the release of the new bacteriophages from the cell. The density of adsorbed phages was averaged over large surfaces (compared to a surface of one cell). In this connection we should stress that local values of *n* may be much higher in the proximity of the new phage particles release from an infected cell.

The increase of the roughness of the infected bacterial cells is directly connected with the phage adsorption on the cell surface and with the damage of the cell wall (MOI is quite high in AFM experiments). During the late phase of the lytic cycle bacterial cell produces peptidoglycane lysing enzymes (endolysins), which destroy cells from within. This leads to a significant increase of the roughness (more than 10 nm). Even being measured in air, the changes in the surface roughness reflects the real changes of the cell wall during the infection process. For example, the roughness of the rippling structure (2.5 nm) may serve as a reference point for comparison of all further surface changes. Average RMS surface roughness of different *A. baumannii* strains measured from AFM images obtained in air, has also served as a characteristic of the cell surface upon its colistin treatment [29].

## Concluding Remarks

Utilization of AFM together with microbiological assessment for investigation of the lytic cycle of bacteriophages gives an opportunity not only to compare data obtained independently, but also visualize infection process at the level of one cell. Studies of the dynamics of *A. baumannii* 1053 infection by phage AP22 have demonstrated that the phage completely lyses a liquid culture of the strain in 60–120 minutes depending on MOI. The presence of expanding with time opaque haloes on bacterial lawn of the strain has indicated phage-induced depolymerase activity. AFM has revealed the morphology of bacteriophages AP22 and *A. baumannii* cells, both alone as well as of infected *A. baumannii* cells at different stages of the lytic cycle, from phage adsorption to the cell lysis. Bacteriophages AP22 showed different apparent diameters of their heads depending on the surface to which they adsorb; however, the diameter of the head of the intact phage can be estimated as 62±1 nm and length of the tail as 88±9 nm. Phage specific adsorption to the host cell took place already during the first minute after phages addition to the cells. Estimation of the latent period of the studied phage and time needed for its specific adsorption, obtained from the analysis of AFM images, was in good agreement with one-step growth experiments [9]. Moreover, AFM has demonstrated strong ability of phage AP22 for fast “proto-pellicles” disaggregation that also suggests AP22- depolymerase activity.

## Materials and Methods

### Phage and Host Strain Propagation

In this work we used the recently described lytic phage AP22 specifically infecting *A*. *baumannii* [9]. Single plaque isolation was used to obtain pure phage stock. For that reason, a single plaque formed on the *A*. *baumannii* lawn was picked up in SM-buffer (10 mM Tris-HCl pH 7.5, 10 mM MgSO_4_, 100 mM NaCl) and replated three times. *A*. *baumannii* strain 1053 was used as the host for phage propagation. The strain was obtained from the State collection of pathogenic microorganisms and cellular cultures «SCPM-Obolensk» (accession number B7129) [9]. The bacterial culture of this strain was grown in Luria-Bertani (LB) broth or Nutrient Agar (Himedia Laboratories Pvt. Limited, India) at 37 °C. Phage AP22 was propagated using liquid culture of *A*. *baumannii* 1053 (OD_600_ of 0.3) at MOI of 0.1. The incubation was performed at 37°C until complete lysis.

### Determination of Infection Parameters

The ability of phage AP22 to infect *A. baumannii* 1053 cells in a liquid culture at different multiplicities of infection (MOI) was estimated using approach like in [41]. Briefly, the optical density at 600 nm of phage propagation *A. baumannii* 1053 strain infected at MOI of 50, 5, and 0.5 at 37°C was determined in definite time intervals.

### Bacterial Cell and Bacteriophage Preparation for AFM Experiments

For AFM experiments beforehand propagated and titrated phages (10^9^ PFU ml^−1^) were used. An overnight liquid bacterial culture of *A. baumannii* 1053 strain incubated at 37°C without shaking was diluted in fresh LB-broth and grown stagnantly during 90 minutes at 37°C. Then, the bacterial culture was divided into control and experimental parts. The control part was incubated without phages and the experimental part was mixed with the phage at MOI of 50. This MOI was used to make interaction of the phage with bacterial host cells more obvious. Aliquots of these samples incubated at 37°C were taken at different times (varying from 1 to 60 min) to investigate the bacteriophage infection process with AFM.

### AFM Sample Preparation and Imaging

Square plates (approximately 7×7 mm) from mica or HOPG (for phages adsorption only) were used as substrates in AFM experiments. Either *A. baumannii* cells or their mixture with phages (sample) taken as described above were diluted 11 times by adding 1 µl of this sample to a milli-Q water droplet (10-µl), deposited directly onto a freshly cleaved mica surface. Then the sample was left for 1 minute for adsorption, gently rinsed from the pipette with 200 µl of Milli-Q water and dried in the laminar flow hood. All specimens were prepared prior to AFM imaging. Nanoscope III (DI, Santa Barbara, USA), equipped with 150-µm scanner and operated in contact mode in air, was used for AFM experiments. Commercial silicon CSC11 cantilevers (MikroMasch) had a typical spring constant of 0.35 N/m. The FemtoScan software (Advanced Technologies Center, Russia) was used for image processing. The scan angle was 90° and the scan rate was typically 2 Hz with 512 lines.

## Supporting Information

Figure S1 AFM image of *A. baumannii* cells infected for 5 minutes. Scan size is 50 µm.
(DOC)

Figure S2 AFM height (left row), deflection (middle row) images and section analysis (right row) along the dotted lines on the corresponding AFM images of *A. baumannii* cells incubated with bacteriophages AP22 for 30 minutes.
(DOC)

Figure S3 AFM height (left) and deflection (right) images of *A. baumannii* cells incubated with bacteriophages AP22 for 60 minutes. Scan size is 4.2 µm.
(DOC)

Figure S4 AFM image of *A. baumannii* cells infected for 60 minutes. Scan size is 13 µm. The height of the infected cells ranges from 50 to 100 nm, indicating that they are destroyed.
(DOC)
