# Supplementary figures and images for: Atomic Force Microscopy Analysis of the Acinetobacter baumannii Bacteriophage AP22 Lytic Cycle
Source: PLoS One. 2012 Oct 11;7(10):e47348. doi: 10.1371/journal.pone.0047348 (PMC3469531; doi:10.1371/journal.pone.0047348)

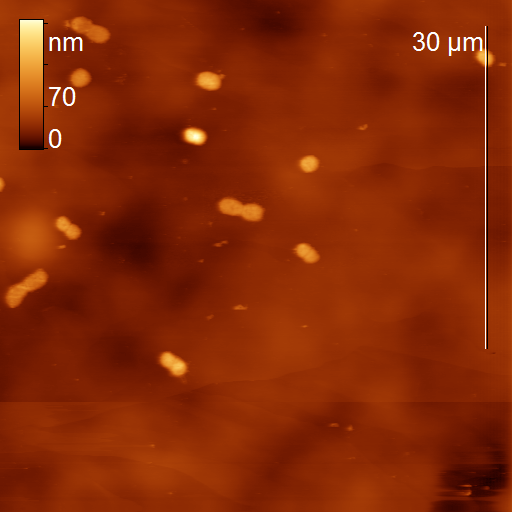

Supplement: Figure S1 — AFM image of A. baumannii cells infected for 5 minutes. Scan size is 50 µm. (DOC) [file pone.0047348.s001.doc]

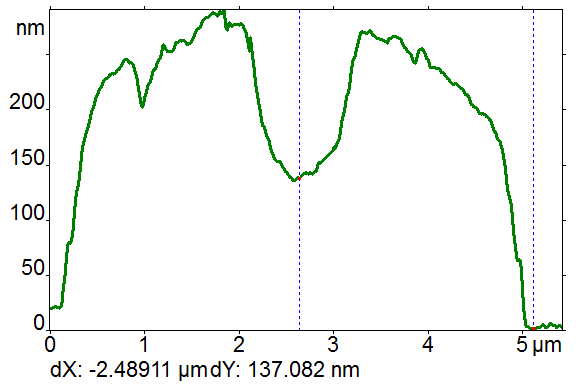

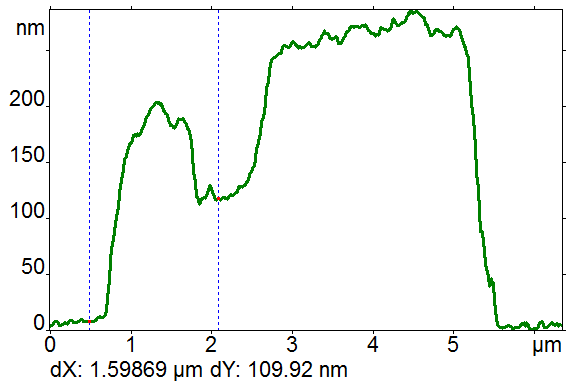

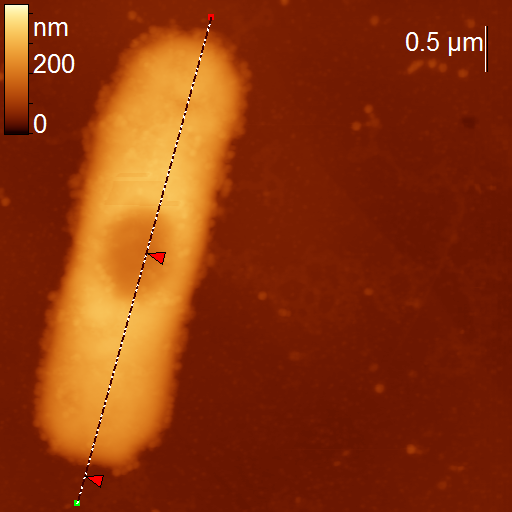

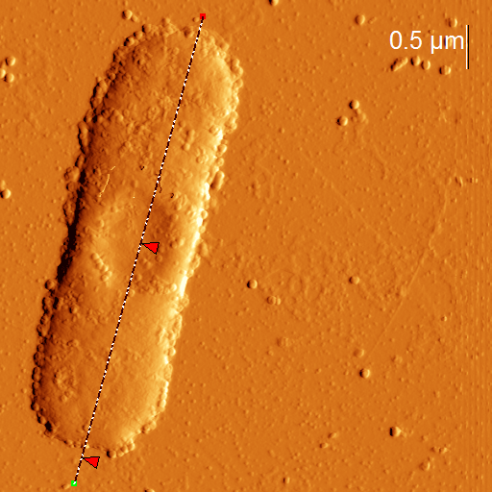

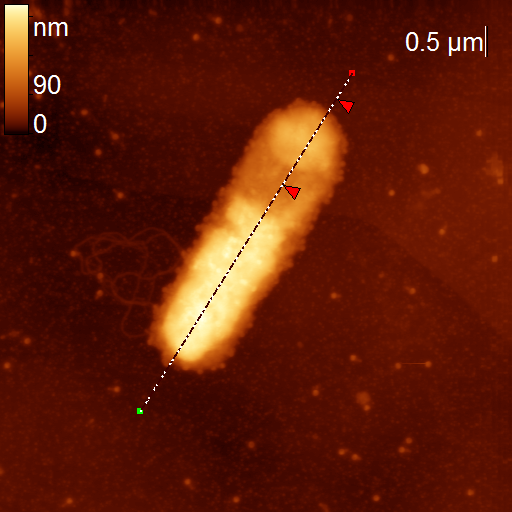

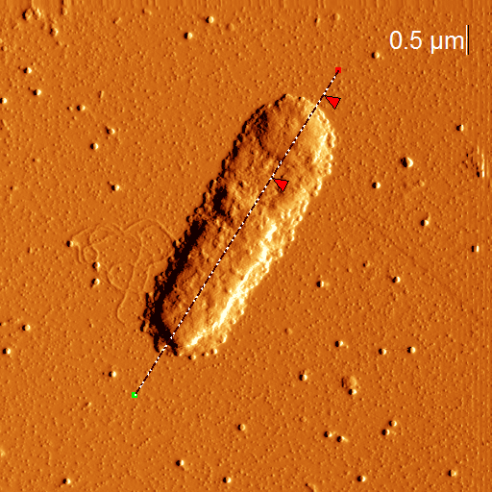

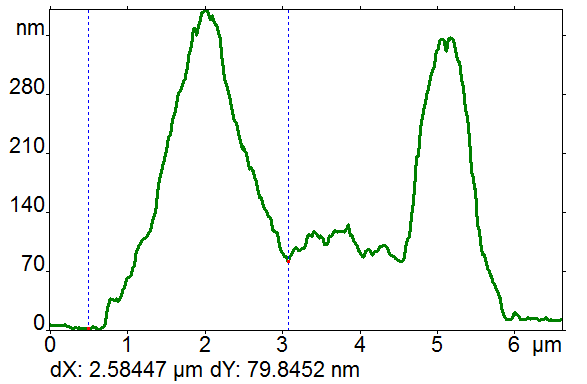

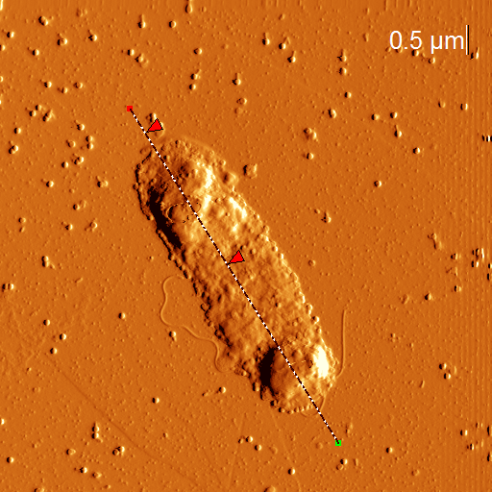

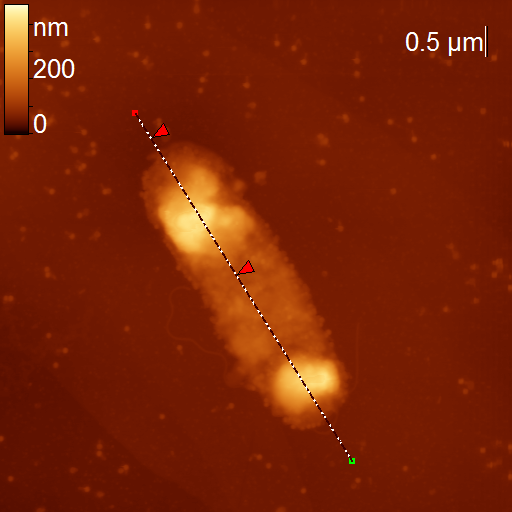


**A**

**B**

**C**

**D**

**E**

**F**

**G**

**H**

**I**

Supplement: Figure S2 — AFM height (left row), deflection (middle row) images and section analysis (right row) along the dotted lines on the corresponding AFM images of A. baumannii cells incubated with bacteriophages AP22 for 30 minutes. (DOC) [file pone.0047348.s002.doc]

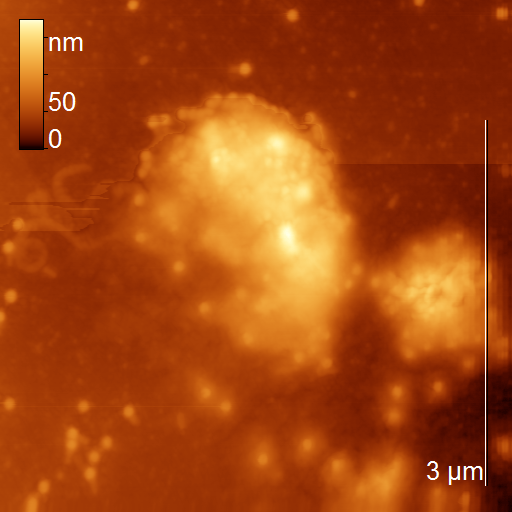

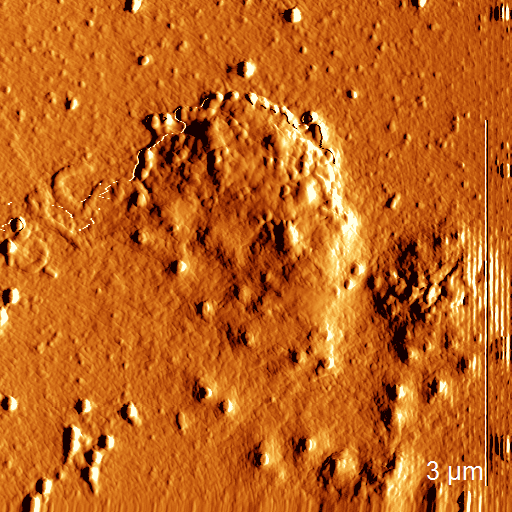

Supplement: Figure S3 — AFM height (left) and deflection (right) images of A. baumannii cells incubated with bacteriophages AP22 for 60 minutes. Scan size is 4.2 µm. (DOC) [file pone.0047348.s003.doc]

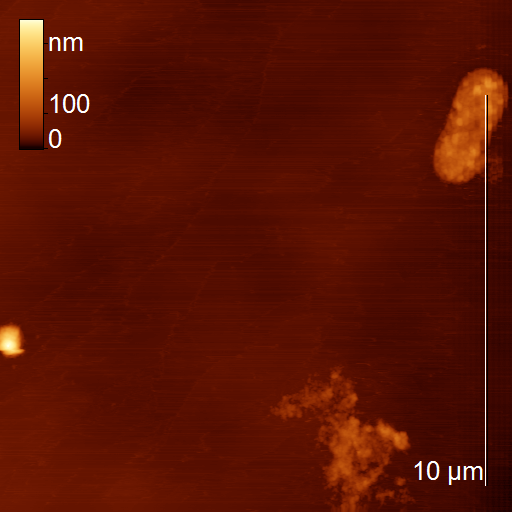

Supplement: Figure S4 — AFM image of A. baumannii cells infected for 60 minutes. Scan size is 13 µm. The height of the infected cells ranges from 50 to 100 nm, indicating that they are destroyed. (DOC) [file pone.0047348.s004.doc]
